# Early term effects of robotic assisted gait training on ambulation and functional capacity in patients with spinal cord injury

**DOI:** 10.3906/sag-1809-7

**Published:** 2019-06-18

**Authors:** Mustafa Aziz YILDIRIM, Kadriye ÖNEŞ, Gökşen GÖKŞENOĞLU

**Affiliations:** 1 Department of Physical Medicine and Rehabilitation, İstanbul Physical Medicine and Rehabilitation Education Research Hospital, İstanbul Turkey

**Keywords:** Robotics, spinal cord, walking, gait, rehabilitation

## Abstract

**Background/aim:**

The aim of the study was to determine the effects of robotic-assisted gait training on ambulation and functional capacity in patients with spinal cord injury.

**Materials and methods:**

In total, 88 patients were included and were randomly divided into two groups. The first group underwent 16 sessions of robotic therapy training for 8 weeks and conventional therapy for 5 days a week. The second group underwent conventional treatment. The Walking Index for Spinal Cord Injury II was used to evaluate functional ambulation, and the functional independence measure score was used to assess patients’ functional independence levels in a blind manner.

**Results:**

A significant improvement was observed in both groups according to Walking Index for Spinal Cord Injury II and functional independence measure scores (P < 0.001). However, a significantly higher improvement according to the Walking Index for Spinal Cord Injury II (P = 0.011) and functional independence measure scores (P = 0.022) was seen in the robotic group than in the control group.

**Conclusions:**

Robotic-assisted gait training combined with conventional therapy was found to be superior to the conventional therapy in terms of gait function and level of disability.

## 1. Introduction

The biggest expectation and concern of patients with spinal cord injuries (SCIs) and their families after an acute period is the regeneration of the gait ability [1]. Therefore, the main strategy in SCI rehabilitation constitutes therapies for improving motor function [2]. The postinjury spinal cord has been reported to be useful for repetitive and relative functional training in terms of self-repair and sensory integration [3]. Studies have shown that repetitive and intensive applications can induce plasticity in the relevant motor centers. Sensory motor stimulation at a sufficient intensity is necessary to optimize neural plasticity. However, since patients are easily fatigued due to severe motor impairment, intensive and repetitive exercises are difficult to perform for a long period, making fatigue an important limiting factor for the conventional rehabilitation program. To overcome this limitation, automatic electromechanical devices have been developed. Robotic-assisted gait training (RAGT) provides many advantages, including maintaining a physiological gait pattern and increasing the intensity of training and the overall duration [4–8]. Robotic systems can be classified as fixed exercise robots for the upper and lower extremities, robots that can be worn on the body (robotic orthoses), auxiliary robots in daily life activities, and robotic walkers. Robots reported in clinical studies ranged from single-jointed simple systems to multimotion systems. Rehabilitation robots can be examined in two groups as end-effector (e.g., Lokohelp, Gait-Trainer 1) and exoskeleton devices (e.g., Lokomat, Robogait) [9–10]. Lokomat has axes aligned with the patient’s anatomical axes and provides direct control of the joints. It reduces the likelihood of abnormal posture and movement. In a preprogrammed walking pattern, the device guides the patient’s legs. It consists of a combination of three joints that can be moved in two hips and one knee with a freely rotatable and two‑dimensional motion pelvis segment [11].

In conventional rehabilitation, while conventional physical therapies, such as stretching, strengthening, and manual-assisted gait training, are practiced by physiotherapists, additional locomotor robotic devices increase the efficiency and performance of physiotherapists [12]. In patients with SCI, beneficial effects on rehabilitation outcomes from locomotor robotic devices cannot be convincingly demonstrated. While some studies show that RAGT is beneficial for improving gait speed, durability, and overall gait ability in patients with SCI, other studies suggest that there is no benefit [13–16]. Moreover, there is insufficient evidence to support the efficacy of RAGT on factors such as gait speed, gait distance, lower extremity motor score, spasticity level, and functional level of independence in patients with SCI[17].

RAGT can facilitate intensive and repetitive exercises without fatigue. Therefore, we hypothesized that RAGT has positive effects on the recovery of ambulation and lowering of disability level in patients with SCI. The aim of this study was to determine the short-term effects of RAGT on the recovery of ambulation and lowering of disability level in these patients. The authors have no financial conflicts of interest.

## 2. Materials and methods

The study protocol was approved by Bakırköy Sadi Konuk Training and Research Hospital Ethics Committee (No. 2018/50). The study was conducted in accordance with the principles of the Declaration of Helsinki. Written informed consent was obtained from each patient.

### 2.1. Inclusion criteria

The inclusion criteria were as follows:

-American Spinal Injury Association Impairment Scale (AIS) patients with SCI with complete and incomplete levels A, B, C, and D

-Patients aged 18–65 years

-Patients whose injury occurred up to 6 months ago

-Patients who could walk independently before the injury

### 2.2. Exclusion criteria

The exclusion criteria were as follows:

-Patients who had previously received robotic therapy

-Severe spasticity in the lower extremity, rigidity, and presence of contracture and fracture

-Presence of severe osteoporosis

-Lower extremity and pelvic pressure ulcers

-Other neurological disorders that affect gait

-Uncontrolled heart disorders, pregnancy, and severe cognitive and/or communicative disorders

### 2.3. Participants

**Figure 1:**
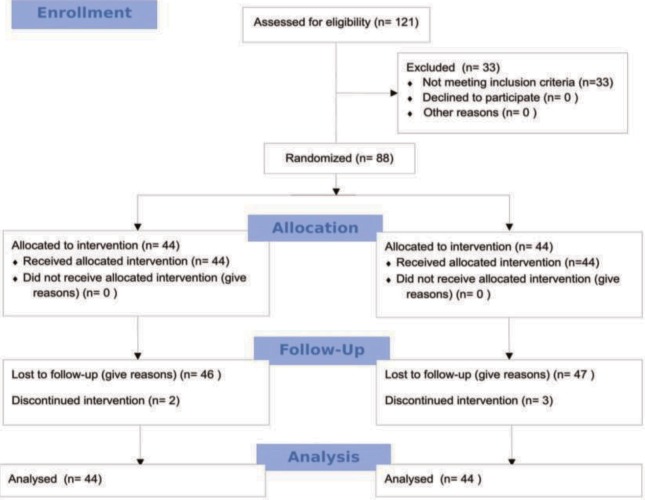
Consort flow diagram.

A total of 121 patients with complete and incomplete SCIs who were treated in the neurological rehabilitation clinic at our hospital were evaluated. Patients with a neurological level of ≥T6 and above were AIS-C and AIS-D. Patients with complete motor and sensory symptoms above the T6 level were excluded from the study due to autonomic problems. The study was completed with a total of 88 patients who met the inclusion criteria (Figure 1).

### 2.4. Treatment protocol

Participants were randomized into 2 groups: the RAGT and control groups. The RAGT group underwent 16 sessions of robotic therapy training for 8 weeks twice a week and conventional therapy (joint range of motion, stretching, strengthening and gait training) for 5 days a week (twice a day). The control group underwent conventional treatment for 5 days a week (twice a day).

Only one investigator was involved in the randomization process, which was performed through coin flipping. The groups were homogeneous in terms of demographic and clinical features (SCI etiology, SCI level, AIS score, and motor level; Table 1).

**Table 1:** Demographic and clinical characteristics of patients.

		Group I (n = 44)	Group II (n = 44)	p
Age (year)		32 (23)	36.5 (24)	0.085*
Duration of disease (month)		3(2)	3(2)	0.482*
Sex	Female	17	16	0.826**
	Male	27	28	
Etiology	Traumatic	34	34	0.999**
	Nontraumatic	10	10	
Motor	Cervical	9	9	0.790**
Level	Lumbar	10	9	
	Thoracic	25	28	
Level	Tetraplegia	9	7	0.580***
	Paraplegia	35	37	
Asia	Complete	21	18	
	Incomplete	23	26	0.520**

Age and disease duration data were given median (interquartile range) Categorical data were given as frequency numbers * Mann–Whitney U test ** Pearson’s chi-square ***Likelihood ratio chi-square Asia: American Spinal Injury Association

### 2.5. Robotic-assisted gait training system

**Figure 2:**
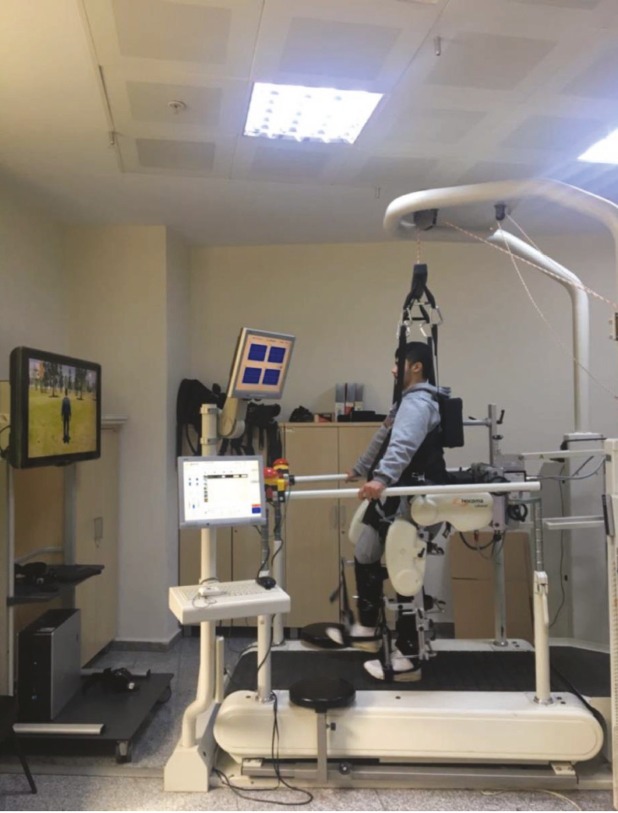
Lokomat, a robot-assisted gait training system.

A computer-controlled exoskeleton system (Lokomat; Hocoma Inc, Zurich, Switzerland) was used for RAGT (Figure 2). The robotic gait orthosis has a force control computer that controls four motors and operates in real time and a feedback monitor that provides motivation and participation to the patient. In addition, the ergonomic structure that holds patients has grip bars, which can be easily adjusted with respect to height and width as well as during treatment. The system and the weight of the patient are taken at the desired amount, and this feature is a dynamic amount that is adjusted in each gait phase of the patient. At the beginning of the gait training, one-half of the body weight was supported, and thereafter the support was reduced. At the end of the rehabilitation program, gait training was completed with full body weight. Patients were gait trained for 30 min in each session. RAGT was performed by a trained physiotherapist.

### 2.6. Evaluation parameters

The patients’ motor impairment was determined using the neurological level and AIS score [18]. The functional ambulation level was evaluated using the Walking Index SCI II (WISCI-II) score, which is a 20-point scale (0 points: no ambulation to 20 points: independent ambulation) [19]. The functional independence levels of the patients were evaluated using the functional independence measure (FIM) score. The FIM includes 13 motor and five social–cognitive measures. The headings include self-care, sphincter care, transfer, locomotion, communication, social interaction, and cognitive activity. A 7-point scale was used to score total independence, wherein 1 indicates complete dependency and 7 indicates full independence. The intermediate levels are 6: modified independent, 5: under supervision, 4: minimal help or >75% of effort consumption, 3: medium-level help or 50%–75% effort consumption, and 2: maximal help or 25%–49% effort consumption [20]. All patients were evaluated by a blind researcher (Kadriye Öneş) at the beginning and end of treatment (single-blind study). The healing rate according to the FIM scale was standardized according to the total FIM score and calculated using the following formula:

Similarly, the healing rate according to the WISCI-II scale was standardized according to the total WISCI-II score and calculated using the following formula:

### 2.7. Statistical analysis

The normal distribution suitability of the variables was tested using the Kolmogorov–Smirnov test with Lilliefors Significance Correction. Quantitative data were represented as median and interquartile range and categorical data as frequency. The distribution of categorical variables to groups was compared using the Pearson chi-square and likelihood ratio chi-square tests. The two groups were compared using the Mann–Whitney U test. The pre- and postrehabilitation averages were compared using the Wilcoxon test for both groups. The SPSS version 18.0 for Windows was used in the analysis. Post hoc power analysis was performed using G*Power version 3.1.9.2 (Franz Faul Universitat Kiel, Germany).

## 3. Results

Demographic data, etiology, AIS score, and motor level are shown in Table 1. There was no statistically significant (P ˃ 0.05) difference between the RAGT and control groups in terms of age; sex; etiology; and percentage of traumatic, nontraumatic, and incomplete patients with spinal cord injury. The FIM score for the robotic group at entry and after treatment was 69 and 85, respectively, whereas for the control group, it was 67 and 77, respectively.

At the end of rehabilitation, a significant improvement was observed in both groups according to the WISCI-II and FIM scores (P < 0.001). The improvement according to the WISCI-II score was significantly higher in the robotic group (5.0%) than in the control group (0%; P = 0.011). Moreover, the improvement according to the FIM score was significantly higher in the robotic group (4.0%) than in the control group (2.0%; P = 0.022; Table 2).

**Table 2:** Comparison of groups in terms of disability level (FIM) and functional ambulation (WISCI II).

		Group I (n = 44)	Group 2 (n = 44)	P-value*
FIM	admission	69.0 (31.0)	67.0 (36.0)	0.576
	out	85.0 (35.0)	77.0 (24.0)	0.118
	P-value**	0.0001	0.0001	
	Healing rate (%)	4.0 (11.1)	2.0 (7.5)	P = 0.022
WISCI II	admission	5.0 (9.0)	5.0 (6.7)	0.521
	out	9.0 (7.0)	6.5 (5.0)	0.028
	P-value**	0.0001	0.001	
	Healing rate (%)	5.0 (38.8)	0(10.0)	P = 0.011

Data were given median (interquartile range) * Mann–Whitney U test / ** Wilcoxon test FIM: Functional Independence Measure WISCI-II: Walking Index Spinal Cord Injury II

### 3.1. Post hoc power analysis

The primary purpose of the study was to examine the effect of robotic rehabilitation on the level of ambulation assessed using the WISCI-II scale. As per the WISCI-II scale, the post hoc power was calculated as 93.1% based on the recovery data: effect size: 0.76, RAGT group mean (standard deviation): 16.9 (20.5), n = 44; control group mean (standard deviation): 5.0 (8.3), n =4 4; and alpha error level: 0.05, bidirectional judgment.

## 4. Discussion

In our study, robotic treatment in patients with SCIs showed a significant improvement in ambulation and functional status. A significant improvement was also observed in the group receiving conventional therapy, but when compared with the group receiving conventional exercise therapy, the recovery in the WISCI-II and FIM values in the RAGT group was found to be more significant. Due to a limited number of randomized controlled trials evaluating the ambulation and functional independence in robot therapy, the effects of robot therapy on walking and functional status in patients with SCI are unclear. Additionally, there is no consensus on the time of treatment initiation and the optimal duration of treatment.

Our study was conducted on complete and incomplete SCI cases in the subacute phase of neurological recovery and a control group with similar age, sex, duration of injury, and lesion level. In a similar study involving complete and incomplete patients, 28 patients were treated with robotic‑assisted treatment for 1 h, 2–3 times per week, but the increase in Functional Ambulation Classification and WISCI scores was not significant compared with that of the conventional group; a significant increase in the Spinal Cord Independence Measure scores was observed [15]. The recovery of motor damages often occurs within the first 2 months after injury and continues in the 3–6-month period. In this period, neuroplasticity continues and is rapid [21]. Our patients were in the first 6 months of motor healing. A total of 16 sessions of RAGT were given to the patients twice a week for 8 weeks. In a systematic review, Morawietz et al. evaluated studies of the acute subacute and chronic periods, in the range of 2–5 sessions per week and of 4–13 weeks in duration; they showed potentials for ambulatory function improvement without superiority compared to another aspect [22]. In two studies using only incomplete patients undergoing RAGT and using parameters similar to the present study, the increase in FIM and WISCI-II scores was found to be more significant compared to the conventional group [14,23].

The limited number of patients, presence of complete and incomplete groups, presence of paraplegic and tetraplegic patients, examination of only early period results, and evaluation with few parameters are the limitations of our study.

In conclusion, we believe that it is beneficial to apply robotic gait training of our study data with other adequate rehabilitation methods and that RAGT is not an alternative but an adjunctive to the conventional therapy. There is still a need for more controlled studies with a larger sample size to assess the different effects of robotic locomotor therapy on different SCI patient populations and to determine the appropriate protocol.
